# Air quality and health risks of residents living near a landfill site in Durban, South Africa

**DOI:** 10.4102/jphia.v16i1.1274

**Published:** 2025-05-31

**Authors:** Phiwayinkosi R. Gumede, Dumile Gumede

**Affiliations:** 1 Teaching and Learning Development Centre, Mangosuthu University of Technology, Durban, South Africa; 2 Faculty of Health Sciences, Durban University of Technology, Durban, South Africa

**Keywords:** air quality, community perceptions, health impacts, landfill site, environmental awareness, South Africa

## Abstract

**Background:**

Landfills are globally recognised as significant environmental and public health risks. Their emissions contribute to air and water contamination. However, research in the South African context remains limited.

**Aim:**

To assess community perceptions of air quality and health impacts of living near a landfill site.

**Setting:**

The study was conducted in Durban, South Africa.

**Methods:**

The study employed a cross-sectional survey design. A structured survey questionnaire was used to collect data from a sample of residents (*n* = 154). Survey interviews were administered in English and isiZulu. Survey data were captured on Microsoft^®^ Excel for descriptive statistical analysis.

**Results:**

The analysis revealed that 72% of respondents rated air quality as poor or very poor, with the landfill site identified as the primary contributor (77%). Seasonal variations were evident, with summer perceived as the season of worst air quality (45%). Awareness of environmental rights and engagement in formal environmental activities were low, with 93% of respondents not participating in any environmental group. Common behavioural responses included shutting windows (60%) and limiting outdoor activities (17%), while 75% of respondents advocated relocation of either the landfill site or the community to address these concerns.

**Conclusion:**

The findings underscore the urgent need for stronger community engagement, targeted awareness campaigns and interventions to address environmental and health challenges near landfill sites.

**Contribution:**

This study advances public health in Africa by highlighting the environmental and health risks of landfill sites and highlighting the need for targeted interventions in affected communities.

## Introduction

Municipal solid waste (MSW) landfills are significant sources of environmental and health risks, particularly for nearby communities. As one of the oldest and most widely used methods of waste disposal globally, landfilling involves burying large quantities of waste in designated ‘dumping cells’, which are then covered with soil daily.^1,2^ While this method has proven effective in managing waste, it also poses considerable risks to both the environment and public health, requiring careful management to mitigate its negative effects.^3^ Over time, landfilling contributes to soil and groundwater contamination, as well as air pollution, resulting in significant social and environmental consequences.^4,5^ Despite its widespread use, the environmental and health risks associated with landfills, such as respiratory diseases, cancer and liver dysfunction, have been well documented, especially for residents living in proximity to these sites.^6,7^ These concerns often include exposure to air pollutants like dust, odour, noise and vermin, which collectively contribute to diminished quality of life for affected communities.^1,8^

The literature on the environmental impacts of landfills reveals that nearby residents are at a higher risk for various health conditions, particularly respiratory problems.^7,9^ Studies on environmental justice further highlight that landfills are often located in economically disadvantaged areas, where marginalised communities bear a disproportionate burden of the associated risks.^10,11^ In developing countries, including South Africa, rapid urbanisation and historical socioeconomic inequalities have exacerbated the vulnerabilities of these communities to the environmental hazards posed by landfills. Problems such as poverty, unemployment, inadequate housing and limited access to essential services heighten the health risks for those living near landfill sites.^10,12^ However, research on landfill-related health and environmental risks in developing nations remains limited, particularly concerning community perceptions of these risks. This gap underscores the need for more comprehensive studies to better understand how local populations respond to the dangers posed by landfill operations.

The operational activities of landfills, including waste transportation, stockpiling, compaction and natural waste decomposition, significantly affect air quality both within the landfill and in surrounding areas.^13^ Emissions from landfills, such as methane (CH_4_), carbon dioxide (CO_2_), particulate matter (PM) and other hazardous volatile organic compounds, contribute to air and water contamination, posing further risks to human health.^1^ Sarker et al.^14^ observed a high incidence of respiratory diseases, cancer and liver dysfunction among residents living near landfills. Similarly, studies in South Africa have linked proximity to landfill sites with an increased likelihood of respiratory problems, especially in children.^15^

Municipal solid waste landfills emit hazardous pollutants, posing significant health risks to nearby communities. Chronic exposure to airborne contaminants, such as particulate matter and toxic gases, has been linked to respiratory conditions, including asthma and bronchitis.^16,17^ These pollutants exacerbate lung inflammation and contribute to long-term respiratory impairment. Additionally, carcinogenic substances like benzene, dichloromethane and heavy metals, including lead, cadmium and mercury, increase the risk of leukaemia, lung cancer and other chronic diseases.^16,18^ Landfill emissions also affect neurological and cardiovascular health. Prolonged exposure to halocarbons and volatile organic compounds (VOCs) can lead to cognitive impairments, headaches and memory loss, while heavy metals contribute to developmental delays in children and neurodegeneration in adults.^17,18^

Cardiovascular issues, such as hypertension and fatigue, are more prevalent among exposed populations.^16,19^ Mental health concerns are increasingly documented, with landfill proximity associated with higher rates of stress, anxiety, depression and sleep disturbances.^19^ Moreover, endocrine-disrupting compounds in landfill emissions have been linked to reproductive issues, including preterm births and congenital anomalies.^16,18^ Mitigating these health risks requires improved landfill management strategies, such as enhanced gas capture systems, stricter waste segregation and comprehensive environmental monitoring. Public health campaigns play a vital role in raising awareness and promoting protective measures for affected communities.^17,18^

Environmental pollution from landfills is also a contributing factor to noncommunicable diseases (NCDs), such as cancer and asthma, as well as birth defects among infants.^20^ Despite the growing awareness of these health effects, there remains a significant gap in research focusing on the perceptions of those living near landfill sites in South African urban centres. This research gap highlights the need for a localised assessment of community perceptions and the evaluation of the air quality and health risks these populations face.

Drawing from studies in developed countries, where community concerns about landfill operations have been well documented,^13^ residents living near landfills are deeply concerned about air pollution, dust and associated health risks. In Malaysia, for example, residents near landfill sites expressed their grievances through media outlets, formal complaints and public protests.^21^ Similarly, in Italy, strong opposition to solid waste treatment and disposal facilities led to protests and calls for the closure of these sites because of concerns over environmental pollution, for example, odour.^8^ These examples highlight the importance of understanding community perceptions of the management of landfill sites.

Community perceptions of landfill risks are shaped by a combination of sociocultural, economic and informational factors, including emotional ties to the land, social norms and access to information.^22^ These perceptions play a crucial role in shaping community responses to environmental hazards and their engagement in risk mitigation efforts. Despite the importance of understanding these perceptions in MSW management, there is a dearth of research on community attitudes towards landfill-related risks in South Africa, which calls for localised studies that can better inform policy and practice.

This study is grounded in the Risk Perception Theory (RPT),^23^ which suggests that individuals and communities interpret risks through a blend of cognitive, emotional and sociocultural factors.^24^ This theoretical framework posits that risk perception is influenced not only by objective assessments but also by values, beliefs and personal experiences. For communities living near landfills, these factors shape their understanding of health risks and influence their advocacy for environmental justice. By applying RPT, this study explores how communities perceive air quality and its implications for their health, offering a nuanced understanding of the sociopolitical dynamics that drive community responses to environmental hazards. This approach is especially relevant in the South African context, where socioeconomic inequalities intersect with environmental health risks.

The purpose of this study was to assess community perceptions of air quality and the associated health risks of living near a landfill site in the eThekwini Municipality, South Africa. By addressing this gap in the literature, the study aims to provide valuable insights that can inform policy interventions, improve public health outcomes and promote environmental justice in communities that are disproportionately affected by landfill operations.

## Research methods and design

### Study design

This study formed part of a broader research project that measured PM2.5 measurements in an indoor environment to assess its concentration levels and its association with the lung function patterns in children aged between 6 years and 12 years residing within a 2 km radius from the Bisasar Road landfill site.^15^ This component of the study employed a quantitative descriptive cross-sectional survey design to assess community perceptions of air quality and health risks associated with living near a landfill site.

### Study setting

The study was conducted in a community near the Bisasar Road landfill site in the eThekwini Municipality, South Africa. This is one of the largest formal waste disposal facilities in the municipality, which operates close to the Clare Estate community in the city. This landfill processes approximately 5000 tonnes of solid waste daily, emitting pollutants such as particulate matter, gases and odours that pose significant environmental and health risks. Established in 1980, it serves as a critical node for MSW management in the eThekwini Municipality. The proximity of the community to the landfill site provided a unique context for examining perceptions of air quality and associated health impacts.

### Population and sampling

The study population consisted of 154 adult household members aged 18 years to 52 years who lived within a 2 km radius of the Bisasar Road landfill site, had children aged 6 years to 12 years in their households and had lived near the landfill site for at least 5 years. A sample of 154 respondents was considered sufficient to identify key trends while ensuring the reliability of descriptive analysis. Studies exploring public perceptions of landfill-related environmental issues utilised comparable sample sizes, demonstrating their effectiveness in capturing diverse community perspectives.^6,7^ Additionally, structured surveys are a widely accepted approach in community-based environmental health research, providing valuable insights into public concerns and experiences.^10^

A representative sample of respondents was selected using a stratified random sampling technique to ensure diversity in age, education and length of stay near the landfill site. Considering the diverse composition of informal settlement communities where the study was conducted, a stratified random sampling approach was chosen for this study. As part of the bigger study, a spiral walk was conducted to profile the community living within a 2 km radius of the landfill site. The main objective for this walkabout was to identify homesteads with children aged 6–12 years. The target population was categorised into four distinct age groups: 0–20 years, 21–30 years, 31–40 years and 41 years and older. To ensure a balanced representation, participants were randomly selected in proportion to the size of each age group, and questionnaires were distributed accordingly. This method aimed to capture a broad range of perspectives within the community.

### Inclusion and exclusion criteria

The inclusion criteria were carefully selected to ensure that participants had direct exposure to the environmental conditions under investigation. Household members who did not meet the following inclusion criteria were excluded:

- Adults aged 18 years and above were included, as they could provide informed perspectives on air quality, health concerns and behavioural adaptations.
- Residents living within a 2 km radius of the Bisasar Road landfill site were selected since proximity increases the likelihood of experiencing landfill-related pollution and its associated risks.
- Households with children aged 6 years to 12 years were considered to align with the broader study, which examined air quality impacts on child health.
- Individuals who had lived in the area for at least 5 years were included to ensure they had substantial exposure to air pollution over time and could provide insights into health risks.

### Data collection methods

Trained research assistants conducted face-to-face structured survey interviews with respondents for a period of 3 months (November 2013 to January 2014). Data were collected using a paper-based and structured survey instrument, which included both closed-ended and Likert scale questions. The survey was designed to gather information on:

- Respondents’ perceptions of air quality (e.g., ratings of air quality and its seasonal variations).
- Perceived health impacts related to air pollution.
- Awareness of environmental rights and reporting mechanisms.
- Behavioural adaptations to mitigate exposure to air pollution.

During the interviews, research assistants recorded respondents’ responses directly onto the structured survey questionnaire. Survey interviews were conducted in English or isiZulu, depending on the participant’s language preference, to accommodate linguistic diversity. Each survey interview session lasted between 45 min and 90 min.

### Variables and measures

The key variables examined in the study included:

- **Perceptions of air quality:** Measured using a 5-point Likert scale (e.g., very poor to very good).
- **Perceived health impacts:** Assessed through binary (yes or no) and multiple-choice questions.
- **Awareness of environmental rights:** Evaluated through direct questioning about knowledge of specific rights and reporting procedures.
- **Behavioural responses:** Measured by the frequency of specific actions taken to reduce exposure to outdoor air pollution.

### Data analysis

Data were initially entered into Microsoft^®^ Excel for organisation and preliminary processing before being analysed using Jeffreys’s Amazing Statistics Program (JASP version 0.19.3), a free and open-source statistical software designed for both descriptive and advanced statistical analysis. JASP was employed to compute frequencies and percentages, summarise categorical data (e.g., environmental concerns and awareness of environmental rights) and calculate means and standard deviations for continuous variables (e.g., air quality ratings), ensuring a comprehensive analysis of the dataset.

### Reliability and validity

This study employed several strategies to enhance the reliability and validity of the findings. For validity, the survey instrument was developed based on existing literature and the theoretical framework, RPT, to ensure that it captured relevant constructs and variables. Also, Likert scale and binary response formats were used to reduce ambiguity and enhance the accuracy of responses. Reliability was enhanced by (1) standardising the survey instrument and administering it consistently across respondents by trained research assistants and (2) inclusion criteria, such as proximity to the landfill site and residency of at least 5 years, ensuring the relevance of respondents’ experiences to the study objective. To ensure data accuracy and consistency, several quality assurance measures were implemented. Initially, data entry was double-checked by the second author to minimise errors and ensure consistency. Additionally, a systematic verification process was conducted where randomly selected entries were cross-checked against original records to identify and rectify discrepancies. The dataset was also screened for anomalies, including outliers and inconsistencies. Where inconsistencies were detected, clarifications were sought from the primary data sources. These measures helped to enhance the reliability of the data and maintain its integrity throughout the analysis process.

### Ethical considerations

Ethical approval was obtained from the Biomedical Research Ethics Committee at the University of KwaZulu-Natal and was received on 12 April 2012. The ethical approval number is BE201/11. Gatekeeper permission to conduct the study was secured from the eThekwini Municipality. The purpose and objectives of the study were clearly explained to all individuals prior to their involvement. Respondents provided written informed consent before data collection began, signifying their voluntary agreement to participate. Respondents were assured that their responses would remain anonymous, with no identifying information included in the analysis or reporting. Confidentiality of all data was maintained by anonymising respondents’ identities, and respondents were informed that their information would only be used for this research. All data were securely stored in password-protected files, accessible only to authorised researchers. All procedures performed in studies involving human participants follow the ethical standards of the institutional research committee and the 1964 Helsinki Declaration and its later amendments or comparable ethical standards. These measures were implemented to uphold participant confidentiality and maintain the integrity of the study.

This study followed ethical guidelines to safeguard the rights of all participants. According to Creswell,^25^ participants are entitled to protection from harm, autonomy in decision-making, privacy and access to necessary services. Additionally, these rights extend to preserving personal dignity and self-respect, ensuring anonymity and maintaining the confidentiality of sensitive information.

## Results

### Demographic characteristics of the study respondents

A total of 154 respondents participated in the study, as shown in Table 1.^15^ The study respondents ranged in age from 20 years or younger to 41 years or older, with 33% of respondents aged 41 years and above constituting the largest age group. This age distribution provided a diverse range of perspectives across different life stages. In terms of educational background, respondents generally had low levels of formal education. Only 26% of respondents reported being graduates of a university, college or vocational and technical college. This demographic characteristic highlights the need to consider varying levels of education in understanding respondents’ perceptions and behaviours. All respondents had resided in the area for a minimum of 5 years, ensuring they had substantial familiarity with and experience with the local environmental conditions. This long-term residence enhanced the reliability of their insights regarding air quality and its impact on the community. This diverse yet locally rooted respondent group provided valuable data reflecting both a range of demographic perspectives and an understanding of the study context.

**TABLE 1:** Demographic characteristics of residents showing ages, educational level and period of stay (*N* = 154).

Variable	Frequency (*n*)	%
**Age (years)**		
≤ 20	11	7.0
21–30	44	29.0
31–40	49	32.0
≥ 41	50	33.0
**Highest education level**		
Nonmatriculants	53	34.0
Matriculants	62	40.0
Vocational school or technical college	7	5.0
College or university graduate	22	21.0
**Length of stay near a landfill site (years)**		
5	37	24.0
6–10	44	29.0
11–15	73	47.0

*Source:* Adapted from Gumede PR, Savage MJ. Respiratory health effects associated with indoor particulate matter (PM2.5) in children residing near a landfill site in Durban, South Africa. Air Qual Atmos Health. 2017;10(7):853–860. https://doi.org/10.1007/s11869-017-0475-y

### Community concerns about environmental impacts

The survey results highlight the community’s environmental concerns, with particular emphasis on matters related to the nearby landfill site. These results are categorised into three: primary environmental concerns, minor concerns and no reported concerns.

**FIGURE 1:**
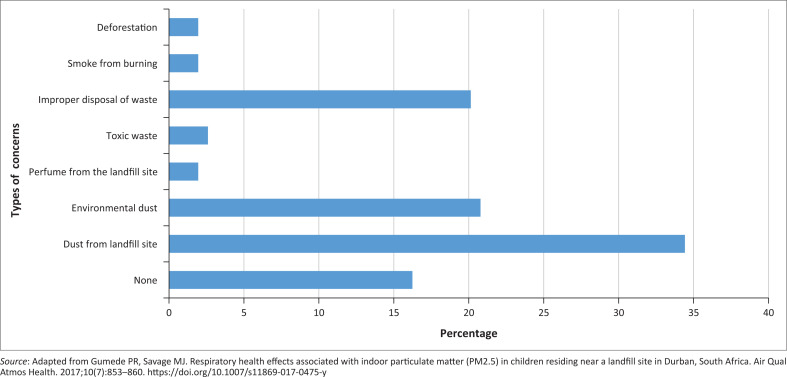
A bar graph showing the greatest environmental concerns of respondents.

Respondents expressed significant unhappiness about the existence of the landfill site, identifying it as the most pressing environmental concern (Figure 1).^15^ The top three primary environmental concerns mentioned by respondents were dust from the landfill site (34%), the environmental dust (21%) and improper disposal of waste at the landfill site (20%). Collectively, these top three concerns account for 75% of all reported concerns, reflecting a strong focus on the landfill site and its impact on air quality and waste management.

Other minor environmental concerns were mentioned less frequently and were categorised as minor. This included deforestation (2%), smoke from burning (2%), perfume or odour from the landfill site (2%) and toxic waste (3%). Together, these minor concerns accounted for 9% of the total concerns reported. However, 16% of respondents indicated that they had no environmental concerns.

To gain further insights from the data, descriptive statistics for local environmental concerns are presented in Table 2.^15^ The analysis of local environmental concerns highlights significant community dissatisfaction, particularly with dust and improper waste disposal at the landfill site. The *mean concern frequency* (*n* = 19) and a high standard deviation (*n* = 19) suggest notable variability in perceptions, while the mode (*n* = 3) and median (*n* = 15) indicate that certain concerns were more common than others. A wide range (*n* = 50) and 95% confidence interval (3–35) further illustrate the disparity in community concerns. Despite 75% of respondents identifying dust and waste management as primary issues, 16% reported no concerns, suggesting varying levels of environmental awareness or adaptation to poor conditions. The findings emphasise the need for targeted awareness campaigns, improved landfill management and stronger community engagement to address environmental and health risks effectively.

**TABLE 2:** Descriptive statistics for local environmental concern.

Descriptive statistics	Frequency (*n*)
Mode	3
Median	15
Mean	19
95% CI mean upper	35
95% CI mean lower	3
Standard deviation	19
Range	50
Minimum	3
Maximum	53

*Source:* Adapted from Gumede PR, Savage MJ. Respiratory health effects associated with indoor particulate matter (PM2.5) in children residing near a landfill site in Durban, South Africa. Air Qual Atmos Health. 2017;10(7):853–860. https://doi.org/10.1007/s11869-017-0475-y

CI, confidence interval.

The results highlight that the community’s primary environmental concerns are closely tied to the landfill site’s operation, particularly those of dust and improper waste disposal, which dominated the environmental discourse. Minor concerns and the lack of concern reported by a subset of respondents indicate varying levels of environmental awareness and prioritisation within the community.

### Perceptions of air quality and seasonal impacts

The survey results provided a comprehensive overview of respondents’ perceptions of air quality in their area over the past 12 months, highlighting concerns about pollution, seasonal variations and contributing factors.

### Air quality ratings

Respondents rated air quality using a Likert scale ranging from very poor to very good. Of the total respondents, 72% rated the air quality as poor, comprising 45% who rated it as very poor and 27% who rated it as poor. A smaller proportion, 11%, rated the air quality positively, whereby 9% rated it as good and 2% rated it as very good. Of the respondents, 17% reported that air quality was neither good nor poor, possibly reflecting mixed perceptions influenced by economic benefits some residents derive from waste-picking activities at the landfill site. The results demonstrated widespread dissatisfaction with air quality in the area, with most respondents perceiving it as poor or very poor.

### Seasonal variation in air quality

Respondents identified seasonal differences in air quality. Winter was cited by 44% as the season with the best air quality, whereas summer was reported by 45% as the season with the worst air quality. These seasonal differences in air quality were solely based on respondents’ experiences and perceptions. These results suggest that seasonal factors, such as weather patterns and atmospheric conditions, play a significant role in influencing air quality in the area. Seasonal variations in air quality further emphasised the dynamic nature of pollution in the community, with worse conditions reported in the summer months.

### Key contributors to poor air quality

Respondents identified multiple contributors to poor air quality in the area, with a strong consensus regarding the primary source of pollution.

#### Landfill site

The landfill site was overwhelmingly cited as the main contributor to poor air quality, with 77% of respondents attributing air pollution in the area to its operations. This aligns with the broader community concerns regarding the landfill’s impact on local environmental and health conditions.

#### Industrial emissions

A 6% of respondents highlighted emissions from industrial operations as a source of air pollution, pointing to localised industrial activities that contribute to deteriorating air quality.

#### Motor vehicle exhaust fumes

Another 6% of respondents identified motor vehicle exhaust fumes as a significant contributor, reflecting concerns about traffic-related air pollution in the area.

#### Dust from residential areas

Another 6% of respondents attributed poor air quality to dust from residential areas, which may be linked to unpaved roads or construction activities.

#### Smoke from agricultural burning

A 5% of respondents mentioned smoke from agricultural burning as a source of air pollution, highlighting periodic practices that contribute to localised air quality concerns.

The results reveal that the landfill site is perceived as the dominant contributor to poor air quality, far surpassing other sources such as industrial emissions, vehicle exhaust fumes, residential dust and agricultural burning.

### Community engagement and awareness in environmental activities

The study measured respondents’ involvement in environmental activities aimed at reducing or mitigating air pollution and their general awareness of environmental rights. The results indicate a significant lack of awareness and engagement among the community.

#### Awareness of environmental rights

About 60% of respondents reported that they were unaware of their environmental rights as citizens of South Africa. This lack of awareness highlights a critical gap in knowledge that may hinder active participation in addressing environmental concerns.

#### Involvement in environmental organisations or groups

The vast majority (93%) of respondents had not been involved in any environmental organisation or group, reflecting minimal community participation in formal environmental initiatives.

#### Participation in environmental campaigns or protests

Many participants (86%) stated that they had never participated in an environmental campaign, project or protest, indicating low levels of activism or engagement in efforts to address air pollution sources.

#### Membership in air quality and public health organisations

An overwhelming 97% of respondents reported that they were not members of any organisation concerned with air quality or public health, further illustrating the lack of structured community involvement in tackling air pollution.

#### Knowledge of reporting mechanisms

Most participants (94%) indicated that they did not know the appropriate contact number to register an air quality complaint. This suggests a lack of access to or awareness of mechanisms for reporting environmental grievances.

Overall, the results reveal a significant gap in community awareness and involvement in environmental protection activities and rights.

### Community actions and proposed solutions to address outdoor air pollution

This section reports on respondents’ behavioural responses to outdoor air pollution and their proposed solutions to address the challenges posed by proximity to a landfill site. In this study, we define behavioural responses as the specific actions respondents take to minimise their exposure to air pollution. The results provide insights into how residents adapted to environmental risks and what they viewed as effective interventions.

### Behavioural actions to reduce exposure to outdoor air pollution

Respondents were asked to indicate the actions they had taken over the last 12 months to reduce their exposure to outdoor pollution. This provided insights into social behaviours influenced by perceptions of air quality. The actions taken and reported by respondents were primarily behavioural and categorised into prominent actions and less common strategies.

#### Prominent actions

The prominence of certain actions, such as shutting windows (reported by 60% of respondents), reflects the widespread perception that controlling indoor air quality is a feasible and immediate approach to mitigating exposure. Additionally, the results show that 19% of respondents reported doing nothing, suggesting a lack of resources or awareness regarding effective mitigation strategies. The decision to limit outdoor activities (17%) further highlights the awareness of air quality issues and an attempt to minimise exposure by adjusting daily routines.

#### Less common strategies

Leaving their homes temporarily to avoid pollution was reported by 4% of respondents. Also, registering air quality complaints with the municipality and skipping a day of work were the least reported actions, each cited by 1% of respondents.

These results highlight a reliance on individual, immediate actions rather than systemic solutions, emphasising the need for structured interventions to support residents in managing air quality challenges.

### Proposed solutions to air pollution problems

Respondents were also asked to suggest solutions to mitigate the environmental and health concerns stemming from the landfill site. Out of 154 respondents, only 42 (27%) abstained from responding, while the remaining 112 (73%) respondents responded. Key proposed solutions included relocation of the landfill or relocation of the community.

#### Relocation of the landfill or community

Relocation was suggested by 84 (75%) of 112 respondents who responded to the question. Of these, 72 (64%) respondents advocated for the closure or removal of the landfill site, while 12 (11%) recommended relocating the community to a less polluted area. The remaining 28 (25%) proposed alternative solutions, which likely represent localised or less widely shared perspectives.

The results demonstrate that respondents were aware of the poor air quality in their area and were taking actions within their means to reduce exposure, such as shutting windows or limiting outdoor activities. However, a significant number of residents felt disempowered, as evidenced by the high percentage of respondents doing nothing or abstaining from proposing solutions. The overwhelming call for the relocation of the landfill or the community highlights the residents’ perception that the root cause of the problem is beyond their control and requires urgent intervention by the municipality.

## Discussion

This study provides a comprehensive understanding of community perceptions, behaviours and proposed solutions regarding environmental and air quality concerns, using the lens of RPT. This theoretical framework highlights how individuals assess risks based on cognitive and emotional factors, shaped by personal experiences and social contexts.^23^ The findings emphasise significant concerns, gaps in awareness and behavioural responses, which collectively inform the recommendations for policy, interventions and future research.

The study reveals that the landfill site is the predominant source of environmental dissatisfaction, with 75% of concerns related to dust, improper waste disposal and general environmental dust. This aligns with RPT, which posits that risks perceived as involuntary and controllable by external entities (e.g., municipalities) often elicit stronger reactions.^23^ Minor concerns, such as deforestation (2%) and smoke from burning (2%), suggest a tiered prioritisation among residents based on perceived immediacy and severity of threats. A notable 16% of respondents reported no environmental concerns, possibly indicating variations in risk awareness or normalisation of adverse conditions because of prolonged exposure. This finding is consistent with previous studies, which indicate that prolonged exposure to environmental hazards can lead to diminished risk sensitivity or a sense of fatalism^24^ and highlighting the need for targeted awareness campaigns to bridge gaps in environmental risk recognition.

A significant majority of respondents (72%) rated air quality as poor or very poor, underscoring widespread dissatisfaction. Interestingly, perceptions of seasonal air quality diverge from objective air quality measurements from other studies. Although the difference is marginal, 44% of respondents associate winter with improved air quality and 45% regard summer as having the worst conditions, objective air quality measurements from other studies contradict these subjective assessments. A study about the dispersion of atmospheric air pollution in summer and winter reports that PM concentration levels peak during winter because of increased emissions from heating activities, whereas summer generally experiences lower pollution levels.^26^ These findings underscore the strong seasonal influence on air pollution, shaped by heating emissions in colder months and meteorological factors affecting pollutant dispersion.

Previous research has identified multiple factors contributing to the discrepancy between subjective perceptions of air quality and objective measurement data. The perception that summer has the worst air quality in Durban is largely influenced by meteorological and environmental conditions.^27^ Increased outdoor activity during the warmer months heightens public awareness of air pollution, making landfill emissions more noticeable and reinforcing negative perceptions.^28^ Additionally, elevated temperatures accelerate waste decomposition and enhance ozone formation by promoting chemical reactions between nitrogen oxides (NOx) and VOCs.^26^ Although winter inversions trap pollutants, their effects may be less visually perceptible compared to the combined impact of humidity and pollution during summer.^29^ Furthermore, stronger onshore winds and sea breezes can aid in pollutant dispersion but may also transport industrial and biomass emissions into the city, influencing air quality dynamics.^26^

According to RPT, perceived risks are often heightened by visible and recurring events, such as seasonal air quality deterioration, which amplifies concerns and emotional responses.^30^ These findings emphasise the dynamic nature of air quality concerns, necessitating adaptive interventions that account for temporal and environmental factors. Similar patterns were observed that seasonal fluctuations^31^ are critical in shaping public perceptions of air quality.

The landfill site was identified as the dominant contributor to poor air quality (77%), significantly overshadowing other sources such as industrial emissions (6%), vehicle exhaust (6%) and agricultural burning (5%). This aligns with RPT’s assertion that sources perceived as localised and proximate evoke greater concern.^32^ The landfill’s prominence highlights its role as both a physical and symbolic representation of environmental degradation in the community. Published findings from the broader study indicate that living near landfill sites is associated with a higher risk of respiratory issues, particularly in children.^15^ Previous research corroborates this finding, highlighting that communities living near landfill sites frequently cite these facilities as major sources of pollution and health risks.^8,13,21^ Addressing such concerns requires targeted interventions that balance waste management needs with community well-being.

Furthermore, the results indicate a significant lack of awareness and engagement in environmental protection efforts. Only 40% of respondents were aware of their environmental rights, and 93% had not been involved in environmental organisations. Furthermore, 94% did not know how to report air quality complaints, illustrating barriers to participation in formal environmental advocacy. The low awareness of environmental rights and formal reporting mechanisms among residents near landfill sites is largely attributed to socioeconomic barriers, including limited access to education, inadequate community engagement and insufficient communication from authorities.^7,10^ Marginalised communities often lack exposure to environmental policies, reducing their ability to advocate for their rights effectively.^14^ The RPT highlights that perceived self-efficacy and trust in institutions influence risk-related behaviours.^33^ The community’s limited engagement may reflect feelings of powerlessness and scepticism regarding the effectiveness of collective action or institutional response. A similar trend was reported that mistrust in authorities significantly hampers community involvement in environmental matters.^34^

To bridge this gap, targeted educational programmes in local languages should be introduced through community centres and schools to ensure the accessible dissemination of information.^6^ Additionally, enhancing the accessibility of reporting mechanisms such as dedicated municipal hotlines and mobile applications can empower residents to actively participate in environmental advocacy.^34^ Strengthening collaboration between municipal bodies and local stakeholders through workshops and town hall meetings can further improve awareness and engagement, fostering a more informed and proactive community.^35^

Respondents’ actions to reduce exposure were primarily individual and immediate, such as shutting windows (60%) or limiting outdoor activities (17%). Less common actions, like filing complaints (1%) or temporarily relocating (4%), suggest barriers to systemic solutions, including a lack of access to resources or information. Proposed solutions overwhelmingly focused on relocation (75%), with 64% advocating the landfill’s closure or removal. This reflects the RPT’s notion that externalising responsibility to authoritative bodies is common when risks are perceived as beyond individual control.^23^ The literature illustrates that community-driven initiatives often centre on relocating landfills or transferring communities to safer environments as primary solutions to localised environmental concerns.^8,35^ These community-driven initiatives reflect the desire to mitigate health and environmental risks by moving hazardous waste sites or vulnerable populations away from areas where they are exposed to pollution, contamination or other environmental hazards.

### Study limitations

This study has several limitations that should be considered when interpreting the findings. Firstly, responses may be influenced by social desirability bias, where respondents may provide answers that they believe are more socially acceptable, rather than reflecting their true behaviours or perceptions. This can potentially skew the data, leading to an overrepresentation of favourable or socially approved responses. While the self-reported data collection method provides valuable insights into personal experiences, it has inherent limitations, including potential social desirability bias and recall inaccuracies. The study employed multiple strategies to mitigate social desirability bias and enhance the reliability of self-reported data. Confidentiality and anonymity were prioritised to encourage honest responses while conducting interviews in English or isiZulu, reducing language-related biases. Structured survey interviews administered by trained research assistants ensured consistency and minimised interviewer influence. The use of closed-ended and Likert scale questions helped limit overreporting of positive behaviours and underreporting of negative experiences. Additionally, behaviour-based questions, such as the frequency of actions taken to reduce exposure to air pollution, provided a more objective assessment of residents’ adaptation strategies, reducing reliance on subjective self-assessments.

Secondly, seasonal variations in air quality may have impacted respondents’ responses, particularly those collected during periods of heightened pollution. As air quality can fluctuate significantly depending on seasonal factors, the responses gathered during these times may not accurately represent the overall experiences of residents throughout the year, potentially limiting the generalisability of the findings.

Thirdly, another limitation of this study is the restricted diversity of the sample. While stratified sampling was used based on age groups, education level and period of stay, other demographic characteristics such as socioeconomic status, gender and race were not explicitly accounted for in the sampling process. By excluding these demographic factors, the study missed the opportunity to provide a more comprehensive understanding of how different groups within the community are affected by landfill-related risks. Gender and race are important determinants of vulnerability and perceptions of environmental hazards, and without this data, the study was limited in its ability to address matters of equity and identify specific community needs. Understanding the intersectionality of these factors is crucial for crafting targeted interventions and policies that are inclusive and responsive to all community members. As a result, the findings may not fully capture the perspectives of all socioeconomic subgroups within the community, potentially limiting the generalisability of the study’s conclusions.

These limitations highlight the need for further research incorporating a more detailed demographic analysis to ensure that findings are more representative and actionable in addressing the diverse challenges faced by communities living near landfill sites.

### Recommendations for interventions

Several interventions are recommended to address the challenges identified. Firstly, community awareness programmes should be implemented to educate residents about their environmental rights and reporting mechanisms while also promoting a better understanding of air quality concerns and their associated health impacts. Community awareness programmes can be designed as public campaigns using media, schools and workshops to educate communities. Additionally, incorporating environmental education into the school and university curricula will promote lasting change.

Secondly, enhanced reporting mechanisms should be established by creating accessible, multilingual platforms for lodging complaints related to air quality, ensuring transparency and providing timely feedback on reported matters. Thirdly, landfill site management should be improved by investing in technologies to reduce dust and waste emissions and conducting regular environmental impact assessments with active community involvement. Fourthly, health-focused interventions, such as providing vulnerable households with air purifiers or protective equipment and establishing health monitoring programmes to track pollution-related conditions, should be prioritised to safeguard public health.

### Recommendations for future research

Future research should focus on several key areas to deepen understanding of community perceptions and responses to landfill-related risks. Longitudinal studies using mixed methods are needed to track changes in perceptions and behaviours over time, providing insights into how risk dynamics evolve. Comparative studies across different communities would help identify common challenges and scalable solutions. In addition, exploring the psychosocial dimensions of trust in institutions and the perceived efficacy of interventions can shed light on how these factors influence community engagement with environmental risks. Finally, research should assess the effectiveness of implemented policies and programmes, enabling the refinement of future initiatives aimed at mitigating the impacts of landfill sites on public health and the environment.

## Conclusion

This study highlights the significant environmental and air quality challenges faced by a community living near a landfill site. Guided by the RPT, the findings show the interplay between perceptions, behaviours and systemic barriers to addressing these challenges. While individual actions provide immediate relief, the community overwhelmingly views systemic interventions such as relocating the landfill as essential. Addressing gaps in awareness, engagement and institutional trust will be critical in developing effective, sustainable solutions to improve air quality and public health.

By informing policies and interventions, this study contributes to a broader understanding of how communities perceive and respond to environmental risks, offering a foundation for future research and action.
